# Effectiveness of programs to promote cardiovascular health of Indigenous Australians: a systematic review

**DOI:** 10.1186/s12939-018-0867-0

**Published:** 2018-09-27

**Authors:** Vainess Mbuzi, Paul Fulbrook, Melanie Jessup

**Affiliations:** 1 Nursing Research and Practice Development Centre, The Prince Charles Hospital, Brisbane, Australia; 2 School of Nursing, Midwifery and Paramedicine, Australian Catholic University, Brisbane, Australia; 3 Adult Intensive Care Services, The Prince Charles Hospital, Brisbane, Australia; 4 Faculty of Health Sciences, University of the Witwatersrand, Johannesburg, South Africa; 5 School of Nursing, Midwifery and Social Work, Faculty of Health and Behavioural Sciences, University of Queensland, Brisbane, Australia

**Keywords:** Cardiovascular disease, Indigenous Australians, Interventions, Systematic review

## Abstract

**Background:**

Indigenous Australians carry a greater burden of cardiovascular disease than other Australians. A variety of programs has been implemented with the broad aim of improving Indigenous cardiovascular health, however, relatively few have been evaluated rigorously. In terms of effectiveness, understanding how to best manage cardiovascular disease among this population is an important priority. The review aimed to examine the evidence relating to the effectiveness of cardiovascular programs for Indigenous Australians.

**Methods:**

PubMed, CINAHL, PsycINFO, Scopus and Web of Science databases were systematically searched for relevant studies, limited to those published in English between 2008 and 2017. All studies that used experimental designs and reported interventions or programs explicitly aimed at improving Indigenous cardiovascular health were considered for inclusion. Methodological quality of included studies was appraised using design-specific Joanna Briggs Institute critical appraisal checklists. Data were extracted using the Joanna Briggs Institute data extraction form and synthesised narratively.

**Results:**

Eight studies met the inclusion criteria and were assessed to be of varying methodological quality. Common features of effectiveness of programs were integration of programs within existing services, provision of culturally appropriate delivery models with a central role for Indigenous health workers, and provision of support processes for communities such as transportation. It was noted however, that the programs modelled the interventions based on mainstream views and lacked strategies that integrated traditional knowledge and delivery of health care.

**Conclusions:**

Very few cardiovascular healthcare programs designed specifically for Indigenous Australians, which had undergone rigorous study, were identified. Whilst the majority of included articles were assessed to be of satisfactory methodological quality, the nature of interventions was diverse, and they were implemented in a variety of healthcare settings. The limited evidence available demonstrated that interventions targeted at Indigenous cardiovascular health and related risk factors can be effective. The results indicate that there are opportunities to improve cardiovascular health of Indigenous people at all stages of the disease continuum. There is a need for further research into evidence-based interventions that are sensitive to Indigenous culture and needs.

**Trial registration:**

Registered with PROSPERO International: CRD2016046688.

## Background

Indigenous Australians experience significant health disparities compared to the non-Indigenous population [1]. Although Indigenous peoples throughout the world suffer health disadvantages resulting in reduced quality of life, when compared to non-Indigenous people, the health disparity among Indigenous Australians is significant. It is characterised by the high prevalence of preventable disease, including cardiovascular disease (CVD), which has contributed to lower life expectancy of this population [2]. In adopting its *Closing the Gap* policy, the Council of Australian Governments (COAG) committed to address this inequality. Targets were set for a range of health and wellbeing indicators with the aim of closing the Indigenous life expectancy gap within a generation [3] Subsequently, many healthcare policies, strategies, and programs have been implemented with the explicit aim to improve Indigenous health.

In this context it is essential to monitor and evaluate the effectiveness of such programs, especially those focused on CVD, which has a higher incidence among Indigenous Australians compared to others [4] and is the leading cause of morbidity and mortality in this population [1]. Recent data show little improvement in equity of health outcomes and extensive disparity persists [1, 5].

Although CVD is largely preventable [6, 7] it is a major contributor to morbidity, mortality and health disparity worldwide [8, 9] and there is substantial Australian evidence of its contribution to higher rates of morbidity and mortality among Indigenous Australians [2]. In 2009, CVD was 4.6 times more prevalent in the Indigenous population than in other Australians [10]. And, in the most recent Australian burden of disease study, CVD accounted for 12% of the total burden of Indigenous disease, and was responsible for 19% of the gap in total health burden disparity between Indigenous and non-Indigenous Australians [1]. In 2012 it was reported that Indigenous people are more than 70% more likely to die from CVD than other Australians [11], and Australian national statistics from 2016 attributed 13% of all Indigenous Australian deaths to CVD [12]. In addition, the risk factors for CVD are disproportionally higher among Indigenous Australians compared to other Australians. The fourth national report (2012–2016) of Indigenous primary health care national key performance indicators revealed that although there has been a reduction in CVD, most primary healthcare organisations recorded the necessary risk factors to enable CVD assessment for fewer than 50% of their clients [13]. Although many risk factors are theoretically preventable, effective health promotion campaigns in the Indigenous context are met with challenges such as geographical isolation contributing to lack of access and poor resource distribution, cultural sensitivity issues [10, 14, 15], and the complexity of disease [16].

While it is crucial to understand the root causes of Indigenous CVD disparity in terms of the social and economic forces that contribute to, or influence the development of risk factors, it is equally important to formulate strategies that are effective for improving Indigenous health. Cardiovascular health promotion interventions encompass areas such as the definition of the cardiac condition in terms of aetiology, diagnosis of the problem, identification of treatment courses, how to deliver services effectively, expected outcomes in terms of improvements, and maintenance strategies to prevent deterioration of cardiovascular health [17, 18]. The goal of such programs should be to reduce cardiovascular risk, identify and manage complications, provide appropriate and timely health care, and provide support to Indigenous people in their efforts to modify their lifestyle and self-manage their cardiovascular health [19, 20].

The disparity between Indigenous and non-Indigenous Australian morbidity and mortality represents an important target for the design and implementation of effective cardiovascular health improvement programs. Opportunities to prevent and enhance treatment and management of CVD for Indigenous people are important if closure of the health gap is to be achieved. In this context it is imperative that effective programs are provided that are best suited to their unique contexts and needs. Programs should take into consideration Indigenous factors that impact health, such as political history and cultural views and beliefs that affect disease understandings. Whilst it is well-recognised that there is much that needs to be done to optimise Indigenous cardiovascular health, at present there is limited evidence of the effectiveness of cardiovascular programs that aim to improve it. Despite the fact that many healthcare programs have been implemented, the majority have been reported descriptively (e.g. [21–23]) and most lack a rigorous approach to evaluation of their effectiveness. Thus, it is unclear which programs have greatest benefit.

An evidence-based summary of the effectiveness of cardiovascular programs for Indigenous people would provide important information to assist with the development and evaluation of future programs. Thus, the aim of this study was to evaluate the effectiveness of interventions focused on the management of cardiovascular health among Indigenous Australians. The review results may guide health professionals and policy makers towards best practices that in turn may help to improve cardiovascular health outcomes for Indigenous Australians. The review question was: How effective are current programs that aim to improve cardiovascular health of Indigenous Australians?

## Methods

### Design

A systematic review was conducted based on Joanna Briggs Institute (JBI) guidelines [24]. Systematic review methodology enables use of rigorous methods to synthesise previous research data in a scientifically sound manner through formulation of a research question; identification, selection, critical appraisal, data extraction and analysis; and presentation of aggregated outcomes of studies included in the review [25, 26]. The protocol for this review was registered with Prospero International [27].

### Eligibility criteria

This review sought to identify studies within the published peer-reviewed literature that focused on implementation of programs designed specifically for Indigenous Australians, aimed at prevention, treatment or management, or rehabilitation of CVD that were published in English between 2008 and 2017. The date range was selected to identify interventions implemented following the launch of the *Close the Gap* campaign [3]. Experimental studies eligible for review included both randomised controlled trials and non-randomised studies that measured outcomes associated with the cardiovascular program implemented. The inclusion criteria were:

- ***Population:*** Indigenous participants, and mixed sample studies with a larger proportion of Indigenous participants.
- ***Intervention:*** interventions reporting an explicit aim of prevention, management/treatment, or rehabilitation of cardiovascular disease among Indigenous Australians
- ***Comparator:*** comparisons between intervention and control group or baseline results for single group pre-test and post-test studies.
- ***Outcomes:*** demonstration of changes in, but not limited to: behavioural risk factors (e.g. increased participation in exercise, rehabilitation, and dietary management), knowledge of cardiovascular disease, and health assessment measures (e.g. decrease in blood pressure, weight loss).
- ***Study design:*** all types of experimental (randomised and non-randomised) designs.
- ***Limits:*** peer-reviewed journals; publication date range 2008 to 2017; English language.

### Search strategy

The search was conducted in two stages. In September 2016 an initial search of CINAHL from 2008 onwards was conducted to estimate the quantity and quality of published articles. This initial search retrieved very few potential articles for inclusion in the review and even fewer randomised controlled trials. Subsequently, librarian colleagues were consulted to assist with refinement of the search terms, to include all experimental (randomised and non-randomised) designs. A comprehensive search strategy was developed, which was implemented from October 2016 to June 2017. It used a combination of MESH terms and text words that was purposely broad to capture the breadth of available studies. The following sources were electronically searched: CINAHL; Pubmed; PsycINFO; Scopus; and Web of Science. A search strategy using all identified keywords with filters was applied for each specific database (see Table 1). A hand search of the reference lists of the selected articles was also conducted.

### Study selection process

The database search results were imported into Endnote™ where duplicates were removed; after which the titles and abstracts were screened by three reviewers to identify studies matching the inclusion criteria. Full texts of potentially relevant studies were retrieved for further assessment against criteria. All outcome data claimed by the original authors to measure the construct of interest were considered eligible for inclusion. Studies were included if they used quantitative measures to examine effectiveness. Mixed method studies were considered if significant quantitative outcomes were reported.

### Data extraction and synthesis

Quantitative data were extracted from the included studies. One reviewer extracted data using the JBI data extraction form for experimental/observational studies [28]. Data extracted included: author details; year of publication; study design; sample and setting; methods; intervention description; outcomes; and comments/conclusions. These were cross-checked by two reviewers for completeness and accuracy.

The results are synthesised using a narrative approach supported with tables. Heterogeneity of the studies in terms of interventions, settings, outcome measurements, and study designs precluded conduction of a meta-analysis. Studies were categorised by type of intervention strategy (prevention, management or treatment, or rehabilitation). The focus was on changes that occurred as a result of implementing an intervention and measurement of its impact or effectiveness.

## Results

### Study selection

**Fig. 1:**
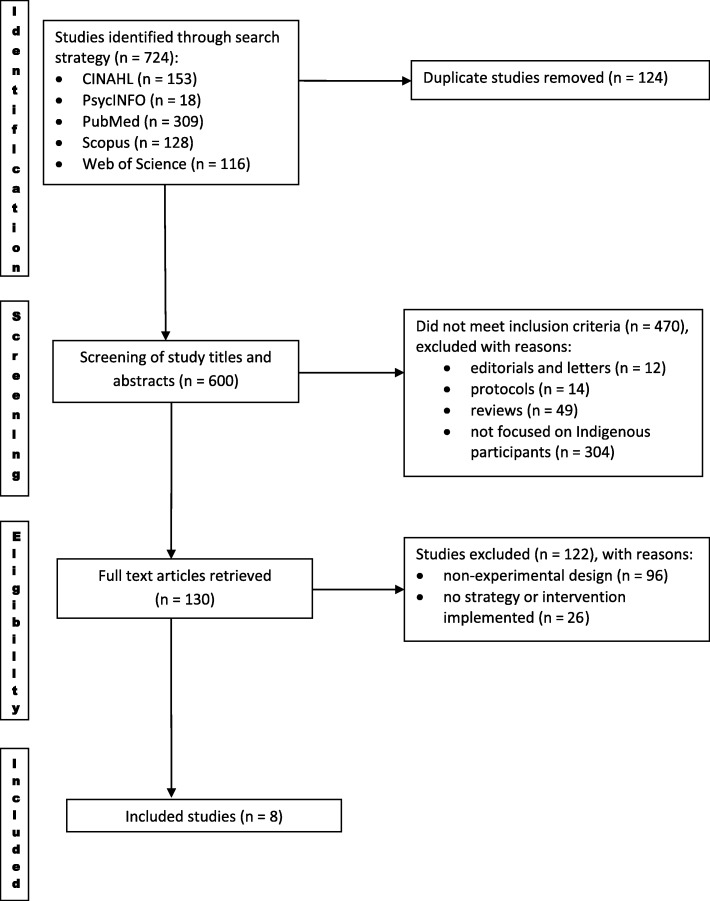
PRISMA flow diagram: search and study selection

The selection process is presented in Fig. 1. A total of 724 studies was retrieved and 124 duplicates were excluded. 470 articles which were: non-specific to Indigenous Australians, qualitative studies, protocols, reviews, editorials or letters; and 122 articles that were purely descriptive or did not involve strategy implementation, were excluded. Eight studies were included in the full review (see Table 2).

**Table 1:** Search strategy in CINAHL

Search	Search terms
S1 Population of interest	((MH “Indigenous Health”) OR (MH “Aborigines+”) OR (MH “Indigenous Peoples+”) OR (Aborigin* OR Indigenous OR “Torres Strait Islander*” OR “Australoid race” OR Koori* OR Murri* OR “Oceanic ancestry group*”))
S2 Disease	((MH “Heart Diseases+”) OR (MH “Coronary Arteriosclerosis”) OR (MH “Coronary Disease+”) OR (MH “Cardiovascular Diseases+”) OR (MH “Cardiovascular Risk Factors”) OR (MH “Cardiovascular Abnormalities+”) OR (MH “Cardiac Patients”) OR (Cardiac OR Cardiolog* OR “Heart disease*” OR “Heart failure*” OR Coronar* OR Cardiovascular OR “Heart health” OR “Heart problem*” OR “Heart disorder*”))
S3 Intervention	((MH “Patient Education+”) OR (MH “Diet+”) OR (MH “Health Services+”) OR (MH “Health Promotion+”) OR (MH “Public Health+”) OR (MH “Epidemiology+”) OR (MH “Treatment Outcomes+”) OR (MH “Rehabilitation+”) OR (MH “Therapeutic Exercise+”) OR (MH “Exercise+”) OR (Prevent* OR Educat* OR Diet* OR Exercise* OR Rehabilitat* OR “Health Promotion” OR “Treatment outcome*” OR “Public health” OR Epidemiology))
S4 Setting	((MH “Australia+”) OR (MH “Western Australia”) OR (MH “South Australia”) OR (MH “Queensland”) OR (MH “New South Wales”) OR (MH “Australian Capital Territory”) OR (MH “Northern Territory”) OR (MH “Tasmania”) OR (Australia* OR Queensland OR “New South Wales” OR Victoria OR “Australian Capital Territory” OR “South Australia” OR “Western Australia” OR “Northern Territory” OR Tasmania) NOT (Canada OR Columbia))
S6 Design	(“Randomized controlled trial*” OR “controlled clinical trial*” OR “random allocation” OR “double blind method” OR “single blind method” OR “clinical trial*” OR placebo* OR random* OR “research design” OR “comparative stud*” OR “evaluation stud*” OR “follow-up stud*” OR “prospective stud*” OR “cross-over stud*” OR (control* OR prospect* OR volunteer*) OR ((singl* OR doubl* OR treb* OR tripl*) W2 (blind* OR mask*)))
S6	S1 AND S2 AND S3 AND S4 AND S5

**Table 2:** Characteristics of included studies

Authors, year	Study design	Strategy focus	Intervention	Sample and setting	Method	Outcomes	Comments
Burgess, et al., 2011 [38]	Interrupted time series study (6 years); pre- and post-measures; 6-monthly follow ups pre- (3 years) and post-intervention (3 years)	Early identification/ preventative	Holistic CVD risk assessment as part of an adult health check	Indigenous participants (*n* = 64); remote primary health care service, Northern Territory	Questionnaires/charts review/ investigations	Improved delivery of preventive care services; improved medicine prescription; reduction in estimated absolute CVD risk; better and earlier identification of elevated CVD risk.	The program led to better and earlier identification of cardiac risk factors as well as generally improved delivery of preventive care services and cardiac treatment
Burgess, et al., 2015 [36]	Longitudinal clinical audits of cardiac prevention services; 3 monthly follow-ups for 2 years	Preventative	Chronic conditions management model	Indigenous participants (*n* = 12,428); remote primary health care services (*n* = 49), Northern Territory	Clinical audit of cardiac prevention services	Increased coverage of Indigenous population CVD risk assessment; assessment of modifiable cardiac risk factors); increased appropriate prescription of medication; achievement of clinical targets for risk reduction	The program demonstrated ability to reduce cardiac risk factors in rural Indigenous populations. It also enabled follow-up of patients
Canuto, et al., 2012 [31]	Pragmatic RCT Follow up 3 monthly (completed: *n* = 59, lost to follow up: *n* = 41)	Management/ treatment	12-week exercise and nutrition program	Indigenous participants (intervention *n* = 51, control *n* = 49); metropolitan area, South Australia	Implementation of a structured exercise program	Reduction in weight and BMI	Low attendance but intervention had positive effects. Requires understanding the barriers to participation
Davey, et al., 2014 [33]	Mixed methods Pre- and post-program measures	Rehabilitation/ secondary prevention	8-week supervised exercise and educational session - cardiopulmonary rehabilitation and secondary prevention program	Indigenous participants (*n* = 92); Aboriginal community-controlled health services, Tasmania	Implemented an exercise and educational program with Indigenous community	Increased participation in rehabilitation; positive changes in health behaviours, functional exercise capacity and health related quality of life. Decreased weight, BMI, and waist circumference Increased 6-min walk test results	Community based interventions have multiple positive impacts
Davidson, et al., 2008 [34]	Mixed methods Pre- and post-measures	Management/ treatment	A partnership model among key education providers, policy makers, non-government organisations, the local area health service and Aboriginal community controlled organisations	Indigenous participants (*n* = 21); metropolitan Technical and Further Education (TAFE), New South Wales	Mixed method evaluation using questionnaires and semi structured interviews	Participants reported increased confidence in ability to provide CVD service to community and demonstrated enhanced CVD knowledge: post-course test mean score 70% vs pre-course score 42%	The model was useful in promoting cardiac knowledge in Aboriginal Health Workers while increasing Aboriginal health knowledge in the mainstream health setting. The model forged partnerships.
Daws, et al., 2014 [37]	Pre- and post -evaluation program	Rehabilitation/ secondary prevention	Working together model of care - (Aboriginal hospital liaison officer and specialist cardiac nurse team)	Aboriginal and Torres Strait Islander participants (*n* = 13); metropolitan tertiary public hospital, Victoria	Retrospective audit	Increased referral rate (15 to 86%) and attendance rate (0 to 62%)	The partnership model approach to care coordination and system changes that were implemented led to improved attendance at cardiac rehabilitation in the participating group
Dimer, et al., 2013 [35]	Mixed methods Pre- and post-program measures	Rehabilitation	Cardiac rehabilitation program; weekly exercise and education sessions	Indigenous participants (*n* = 48); Aboriginal medical services, Western Australia	Evaluation of exercise and educational program	Decreased weight, BMI, BP, waist girth; improved 6-min walk test	Aboriginal Medical Service based cardiac rehabilitation proved to be effective in improving attendance, and cardiac risk factor and health management
Peiris et al., 2015 [32]	Parallel arm cluster-randomized controlled trial	Management/ treatment	Computer-guided quality improvement intervention	Aboriginal and Torres Strait Islander participants (*n* = 38,725); Australian primary healthcare centres (*n* = 60 services), New South Wales and Queensland	Implementation of computerised screening and management algorithm	The intervention was associated with improved overall risk factor measurements	There was minimal support required to implement the tool and had positive effects on improving cardiac risk measurement

*BMI* body mass index, *BP* blood pressure, *CVD* cardiovascular disease

### Methodological quality of included studies

Studies selected for inclusion in the review were assessed for methodological quality. Three researchers reviewed and assessed each study for quality and relevance, using tools appropriate to its design. Joanna Briggs Institute [29] checklists for randomised control trials (13 criteria) and quasi-experimental non-randomised studies (9 criteria) were used. Overall quality was graded using categories cited by Reilly et al. [30] in relation to the proportion of criteria met (poor < 50%, moderate 50–80%, good > 80%), but was not used to exclude studies. Any disagreements that arose between reviewers were resolved through team discussion.

The overall weakness of the included studies was a lack of randomised trials; only two studies were found: a pragmatic randomised trial [31] and a parallel arm cluster randomised controlled trial [32]. The other six studies were of various designs, of which three employed mixed methods [33–35]. The two randomised studies were judged to be of moderate quality (see Table 3), while four of the quasi-experimental studies were judged to be of good quality, with one assessed as moderate [36] and another as poor [37] (see Table 4).

**Table 3:** Critical appraisal of randomised controlled trials

JBI checklist criteria (potential bias)	Studies	
	Canuto et al., 2012 [31]	Peiris et al., 2015 [32]
1. Was true randomization used for assignment of participants to treatment groups? (selection bias)	Yes	Yes
2. Was allocation to treatment groups concealed? (selection bias)	No	No
3. Were treatment groups similar at the baseline? (selection bias/design bias)	Yes	Yes
4. Were participants blind to treatment assignment? (performance bias)	No	No
5. Were those delivering treatment blind to treatment assignment? (performance/detection bias)	No	No
6. Were outcomes assessors blind to treatment assignment? (ascertainment bias)	No	Yes
7. Were treatments groups treated identically other than the intervention of interest? (systematic difference/containment bias)	Yes	Yes
8. Was follow-up complete, and if not, were strategies to address incomplete follow-up utilized? (attrition bias)	Yes	Yes
9. Were participants analysed in the groups to which they were randomized? (intention to analysis)	Yes	Yes
10. Were outcomes measured in the same way for treatment groups? (instrumentation/testing effects threats)	Yes	Yes
11. Were outcomes measured in a reliable way? (measurement bias)	Yes	Yes
12. Was appropriate statistical analysis used? (performance/detection bias)	Yes	Yes
13. Was the trial design appropriate, and any deviations from the standard RCT design (individual randomization, parallel groups) accounted for in the conduct and analysis of the trial? (design bias)	Yes	Yes
Total (%) and quality rating^a^	9/13 (69%) Moderate	10/13 (77%) Moderate

^a^Good: at least 80%, moderate: 50–80%; poor: less than 50%

**Table 4:** Results of critical appraisal of quasi-experimental studies

JBI checklist criteria (potential bias and threat)	Studies					
	Burgess et al., 2011 [38]	Burgess et al., 2015 [36]	Davey et al., 2014 [33]	Davidson et al., 2008 [34]	Daws et al., 2014 [37]	Dimer et al., 2013 [35]
1. Is it clear in the study what is the ‘cause’ and what is the ‘effect’ (i.e. there is no confusion about which variable comes first)? (causation/reverse causation)	Yes	Yes	Yes	Yes	Yes	Yes
2. Were the participants included in any comparisons similar? (selection bias)	Yes	Yes	Yes	Yes	Yes	Yes
3. Were the participants included in any comparisons receiving similar treatment/care, other than the exposure or intervention of interest? (history threat/systematic difference/ contamination bias)	Yes	Yes	Yes	Yes	Yes	Yes
4. Was there a control group? (measurement bias)	No	No	No	No	No	No
5. Were there multiple measurements of the outcome both pre and post the intervention/exposure? (maturation threat, regression to the mean)	Yes	No	Yes	Yes	No	Yes
6. Was follow-up complete, and if not, was follow-up adequately reported and strategies to deal with loss to follow-up employed? (attrition bias)	Yes	Yes	Yes	Yes	Yes	Yes
7. Were the outcomes of participants included in any comparisons measured in the same way? (instrumentation/testing effects threats)	Yes	Yes	Yes	Yes	No	Yes
8. Were outcomes measured in a reliable way? (detection/instrument/measurement bias)	Yes	Yes	Yes	Yes	No	Yes
9. Was appropriate statistical analysis used? (performance/detection bias)	Yes	No	Yes	Yes	No	Yes
Total (%) and quality rating^a^	8/9 (88%) Good	6/9 (67%) Moderate	8/9 (88%) Good	8/9 (88%) Good	4/9 (44%) Poor	8/9 (88%) Good

^a^Good: at least 80%, moderate: 50–80%; poor: less than 50%

With the exception of two studies [32, 36] samples sizes were small (< 100). Two studies compared different cohorts before and after the intervention [36, 37] whereas the remainder reported repeated measures. Two studies did not report any statistical significance of outcomes [36, 37] and one [33] reported several outcomes as being “statistically significant” but did not cite associated values. Only one study [33] reported effect sizes, but did not provide associated significance values, although it was stated in the text that the outcomes were not statistically significant.

### Study characteristics

Considerable heterogeneity of cardiovascular health programs and settings was represented in the eight included studies (see Table 2), which were conducted within the following Australian states and territories: New South Wales [34]; New South Wales and Queensland [32]; Northern Territory [36, 38]; South Australia [31]; Tasmania [33]; Victoria [37]; and Western Australia [35]. The studies were conducted in several settings: Aboriginal medical services [33, 35]; metropolitan [31, 34, 37] and primary care [31, 36, 38].

Intervention categories were identified following completion of data extraction. There were three strategic intervention foci observed in the studies reviewed: prevention; management or treatment; and rehabilitation (see Table 2). All studies reported some statistically significant positive impacts, which were demonstrated by post-intervention improvements to varying degrees. The main outcomes measured were: increased participation [33, 37], reduction in physiological indicators, such as blood pressure and body weight [31, 35, 36], increased confidence of Indigenous staff [34] and overall improvements in identification and management of cardiac conditions [32, 36, 38].

### Risk of bias within studies

Bias was distinguished from quality and reflected within the quality appraisal tools (see Tables 3 and 4). The included studies were assessed for six main domains; selection bias (randomisation), performance bias (blinding of participants/personnel), detection bias (blinding of outcome measures), attrition bias (incomplete outcome data), reporting bias (selective reporting) and other sources of bias [39] using the JBI tool quality appraisal tool [29] as used by Omura et al. [40].

The two randomised studies [31, 32] addressed the risk of bias through randomisation, and ensuring that treatment groups were similar at baseline (selection bias) and both specified the randomisation procedures used [computer Programs for Epidemiologists (PEPI) and permuted block procedure, respectively]. Both studies addressed performance bias by using appropriate statistical analyses for their studies, but neither blinded those providing treatment or participants. The six other studies had no randomisation allocation. Four were pre- and post-test single group designs, one was a before and after study [37], and one used a longitudinal design but made comparison to a pre-intervention population [36]. On the whole, the non-randomised studies used the same measures before and after exposure to the program, on the same participants, and appropriate statistical analyses were used. However, in one study [36] virtually all outcome measures were post-intervention only, with no statistical analyses undertaken.

The two randomised studies were assessed at level 1.c evidence according to JBI levels of evidence for effectiveness of experimental designs, and the six non-randomised studies were assessed at level 2.d evidence [41].

### Risk of bias across studies

Risk of bias across studies was conducted to address the following: risk of measurement, detection, attrition, and selection bias as reported earlier (see Tables 3 and 4). Randomisation was used to assess selection bias for the two randomised trials; both described procedures for randomisation. Due to the nature of the programs, blinding of those who delivered the program and participants was not possible. One study used a single-blinded design in which outcome assessors were blinded [32].

### Results of individual studies

Significant results of individual studies are presented in Table 5. Meta-analysis was not possible due to heterogeneity of the studies.

**Table 5:** Significant results of individual studies

Author year Study design	Sample size *n*	Model or test used	Outcomes			Significance *p*	Effect size
			Measure		Result		
Canuto et al., 2012 RCT [31]	*n* = 100 Exp: *n* = 51 Con: *n* = 49	Regression analysis; adjusted for all potential confounders Repeated measures: baseline (T1); immediate post-program (T2); 3 months post-program (T3)	T1-T2 mean weight change (kg) T1-T3 mean weight change (kg)		−1.65 (95% CI -3.27 - -0.03) −2.50 (95% CI-4.46 - -0.54)	0.046 0.013	NR
			T1-T2 mean BMI change (kg/m^2^) T1-T3 mean BMI change (kg/m^2^)		−0.66 (95% CI -1.27 - -0.05) −1.03 (95% CI -1.79 - -0.27)	0.035 0.009	NR
Peiris et al., 2015 RCT [32]	*n* = 38,725 (60 clusters) Exp: *n* = 19,385 (30 clusters); HR cohort *n* = 5392 Con: *n =* 19,340 (30 clusters); HR cohort *n* = 4916	Repeated measures Generalized estimating equations	Appropriate screening for CVD (%)		Exp: 62.8 Con: 53.4	0.02	RR 1.25 (95% CI 1.04–1.50)
			CVD risk screening small service (< 500) (%)		Exp: 59.8 Con: 37.3	0.02	1.62 (95% CI 1.17–2.26)
			HR cohort: antiplatelet medication prescription escalation (%)		Exp: 17.9 Con: 2.7	< 0.001	RR 4.80 (95% CI 2.47–9.29)
			HR cohort: lipid-lowering medication prescription escalation (%)		Exp: 19.2 Con: 4.8	< 0.001	RR 3.22 (95% CI 1.77–5.88)
			HR cohort: BP-lowering medication prescription escalation (%)		Exp: 23.3 Con: 12.1	0.02	RR 1.89 (95% CI 1.08–3.28)
			HR cohort: proportion obtaining guideline BP targets (%)		Exp: 61.0 Con: 55.0	0.05	1.10 (95% CI 1.00–1.20)
Burgess et al., 2011 [38] Quasi	*n* = 64	Repeated measures *t*-test, McNemar’s test, ANOVA	Delivery of CVD preventive services (%)		Baseline: 30 6-month post health check: 53	< 0.001	NR
			Proportion of evidence-based CVD services delivered (%)		Baseline: 29 6-month post health check: 57	< 0.001	NR
			Prescription of all CVD related medication (%)		Baseline: 28 6-month post health check: 89	< 0.001	NR
			Prescription of anti-platelet medication (%)		Baseline: 4.7 6-month post health check: 68.3	< 0.001	NR
			Prescription of lipid-lowering medication (%)		Baseline: 6.3 6-month post health check 65.1	< 0.001	NR
			Prescription of ACEi/ARB medication (%)		Baseline: 25.0 6-month post health check: 63.5	< 0.001	NR
			Prescription of oral hypoglycaemic medication (%)		Baseline: 17.2 6-month post health check: 33.3	0.04	NR
			Number of cigarettes smoked per day (*n* = 41)		At health check: 3.5 At health check review: 2.6	< 0.001	NR
			Waist circumference (cm) (*n* = 56)		At health check: 98.3 At health check review: 96.4	0.04	NR
			HDL cholesterol (mmol/L) (*n* = 58)		At health check: 1.01 At health check review: 1.11	0.001	NR
			Ratio of total to HDL (*n* = 58)		At health check: 5.7 At health check review: 5.0	< 0.001	NR
			Expected post health check 5-year CVD risk (%) (*n* = 58)		At health check: 3.5 At health check review: 2.6	< 0.001	NR
			Expected post health check 10-year CVD risk (%) (*n* = 58)		At health check: 9.5 At health check review: 10.2	< 0.001	NR
			Mean estimated absolute 10-year CVD risk (*n* = 58)		Expected at health check review: 10.2 Observed at health check review: 8.2	0.004	NR
Burgess et al., 2015 [36] Quasi	Population: 49 primary health care services (*n* = 12,428) Sample: CVD risk assessment documented (*n* = 7266) HR cohort (*n* = 2586)	Post intervention descriptive measures No inferential statistical analyses	CVD risk assessment: proportion of population (%)		Pre: 26.0 (*n* = NR) Post: 58.5 (*n* = 12,428)	NR	–
			HR cohort (*n* = 2586): BP assessment (%)		Post: 93.3 (*n* = 2414)	NR	–
			HR cohort (*n* = 2586): medication prescription (%)		Post: 66.8 (*n* = 1728)	NR	–
			HR cohort (*n* = 2414): achieved BP treatment targets (%)		Post: 56.6 (*n* = 1366)	NR	–
			HR cohort (*n* = 2586): lipids assessment (%)		Post: 96.5 (*n* = 2496)	NR	–
			HR cohort (*n* = 2586): lipid lowering medication prescription (%)		Post: 54.8 (*n* = 1416)	NR	–
			HR cohort (*n* = 2496): achieved lipids treatment targets (%)		Post: 39.6 (*n* = 989)	NR	–
			HR cohort (*n* = 2340): non-smoking status (%)		Post: 50.0 (*n* = 1170)	NR	–
Davey et al., 2014 [33] Quasi	*n* = 72	Repeated measures *t-*test	Weight change (kg)		−0.8 (95% CI -.0.01 - -1.6)	NS – value NR	Cohen’s *d* = 0.04
			BMI change (kg m^−2^)		−0.3 (95% CI -0.01 - -0.06)	NS – value NR	Cohen’s *d* = 0.04
			Waist circumference change (cm)		−3.6 (95% CI -2.5 - -4.7)	NS – value NR	Cohen’s *d* = 0.22
			Incremental Shuttle Walk Test change (m)		106.2 (95% CI 79.1–133.2)	NS – value NR	Cohen’s *d* = 0.11
			6 Minute Walk Test change (m)		55.7 (95% CI 37.8–73.7)	NS – value NR	Cohen’s *d* = 0.11
			Timed up and go test change (sec)		−0.8 (95% CI -0.5 - -1.1)	NS – value NR	Cohen’s *d* = 0.11
			Quality of life (SF 36) change	General health	9.7 (95% CI 4.4–14.9)	S – value NR	NR
				Bodily pain	7.4 (95% CI 0.5–14.4)	S – value NR	NR
				Vitality	15.3 (95% CI 9.6–21.1)	S – value NR	NR
				Social functioning	8.5 (95% CI 0.8–16.3)	S – value NR	NR
				Role emotional	13.5 (95% CI 1.0–26.1)	S – value NR	NR
				Mental health	14.2 (95% CI 8.6–19.9)	S – value NR	NR
Davidson et al., 2008 [34] Quasi	*n* = 17	Repeated measures *t*-tests MANOVA	Knowledge score (range 1–25)		Pre: 9.93 (SD 4.02) Post: 17.43 (SD 3.32)	< 0.001	NR
			Confidence in knowledge score		Pre: 4.46 (SD 1.84) Post: 8.08 (SD 1.60)	< 0.001	NR
			Confidence in skills score		Pre: 4.29 (SD 2.75) Post: 8.16 (SD 1.84)	< 0.001	NR
			Confidence in communication score		Pre: 5.52 (SD 2.39) Post: 8.34 (SD 1.69)	< 0.001	NR
Daws et al., 2014 [37] Quasi	Pre: *n* = 68 Post: *n* = 13	Retrospective audit Descriptive analysis. No inferential statistical analyses	Referral for cardiac rehabilitation (%)		Pre (*n* = 68): 14.7 (*n* = 10) Post (*n* = 15): 86.7 (*n* = 13)	NR	–
			Attendance for cardiac rehabilitation (%)		Pre (*n* = 10): 0 Post (*n* = 13): 61.5 (*n* = 8)	NR	–
Dimer et al., 2013 [35] Quasi	Population *n* = 98 Sample *n* = 48	Repeated measures *t*-test	BMI (kg m^−2^)		Pre: 34.0 (SD 5.1) Post: 33.3 (SD 5.2)	< 0.05	NR
			Waist girth (cm)		Pre: 112.9 (SD 13.6) Post: 108.6 (SD 13.2)	< 0.01	NR
	Sample subset (program completion) *n* = 28		Systolic BP (mm Hg)		Pre: 135 (SD 20) Post: 120 (SD 16)	< 0.01	NR
			Diastolic BP (mm Hg)		Pre: 78 (SD 12) Post: 72 (SD 5)	< 0.05	NR
			6 Minute Walk Test distance (m)		Pre: 296 (SD 115) Post: 345 (SD 135)	< 0.01	NR

*ACEi* angiotensin converting enzyme, *ARB* angiotensin receptor blocker, *BMI* body mass index, *BP* blood pressure, *Con* control, *CVD* cardiovascular disease, *Exp* experiment, *HDL* high density lipoprotein, *HR* high risk, *NR* not reported, *Quasi* quasi-experimental study, *RCT* randomised controlled trial, *RR* relative risk, *S* significant, *SD* standard deviation

### Description of studies

Two studies were specifically focused on preventative programs [36, 38], providing a combination of primary and secondary prevention interventions, including CVD risk assessment [36], and holistic risk assessment [38]. The main outcomes measured included coverage of CVD risk assessment for the Indigenous population, appropriate prescription of medication, achievement of clinical targets (such as reduction of blood pressure and weight), and exercise tolerance.

Implementation of CVD risk assessment opportunities in remote Indigenous communities was conducted in the Northern Territory using a holistic approach as part of an adult health check in a primary health care service [38]. The intervention provided a ‘one-stop-shop’ for participants that included provision of transport services to facilitate access. The aim of this six-year interrupted time series study was to identify whether risk assessment led to better identification of elevated CVD risk, improved delivery of preventative services, and improved the CVD risk profile of participants. The results demonstrated that adult health checks were effective in the early identification of CVD with 24.9% (*n* = 75/301) of patients identified as having elevated risk, of whom 64 participated in the study. Compared to baseline, significant improvements in CVD-related medication prescription rates (*p* <  0.001) were observed at 6-month follow-up. As well, significant improvements in CVD risk factors were demonstrated at health check review follow-up (on average, around a year following initial health check): waist circumference reduction (*p* = 0.04) and HDL cholesterol reduction (*p* = 0.001), with significant reduction in expected versus observed mean estimated 10-year CVD risk (*p* = 0.004). A high level of engagement of Indigenous participants was observed, with the majority undergoing care planning (98%) and pharmacotherapy (89%) by the study end.

In a longitudinal study, conducted over 2 years, a *Chronic Conditions Management Model* was implemented, based on early recognition of CVD [37]. The model focused on improving the prevention, early detection and management of chronic conditions by introducing strategies such as an information system using an electronic health record offering easy access patients, cardiovascular risk assessments, structured care pathways, and standardised treatment manuals. Although CVD prevalence increased with age, 9% of those aged between 20 to 34 years were found to have a high level of cardiovascular risk. Subjects aged 75 years or more were excluded from the analyses as they were categorised as high risk by default. Due to the relatively young age of Indigenous people, the majority of participants were aged less than 45 years. Following implementation, the Indigenous population coverage of cardiovascular risk assessment doubled from 26 to 58.5%. Whilst post-intervention outcomes were reported (medication prescription, and CVD clinical targets, such as blood pressure), no comparisons were made with pre-intervention counterpart measures, thus it is difficult to judge the significance of the reported results.

Three studies addressed management or treatment interventions [31, 32, 34]. A pragmatic randomised study in a South Australian urban setting focused on the effects of a structured exercise and nutrition program [31]. Through education, participants were equipped with relevant knowledge to facilitate self-management of their condition and reduce complications. The program aimed to evaluate the effectiveness of a twelve-week structured exercise and nutrition program in a cohort of urban Indigenous Australian women on waist circumference, weight and biomedical markers of metabolic functioning. Participants were randomly assigned to an experimental ‘active’ group or a ‘waitlisted’ control group. Although the sample size was small (*n* = 100), statistically significant outcomes were demonstrated in weight reduction (*p* = 0.046; 0.013) and associated body mass index reduction (*p =* 0.035; 0.009), over time. Although small reductions in blood pressure were achieved (1.24 mmHg, 2.46 mmHg), though considered clinically significant, they were not statistically significant (although incorrectly reported in the abstract as such).

A parallel arm cluster randomised controlled trial was conducted in 60 Australian primary healthcare centres, which implemented a computer-guided tool for the management and treatment of CVD in the Indigenous population [32]. The tool was effective due to the provision of systematic, step-by-step guidance; practitioners were able to manage patients more effectively and efficiently. And, although the intervention achieved positive outcomes (improved CVD risk screening, *p* = 0.02; improved CVD-related medications escalation, *p* <  0.001 to 0.02), prescription rates per se did not improve, and effectiveness was limited by the availability of sites with computer access. CVD risk screening was significantly better in smaller healthcare centres (< 500 patients) than larger ones (*p* = 0.02).

Davidson et al. [35] sought to improve the confidence of Aboriginal Health Workers in their ability to provide CVD services to their communities. They implemented an educational program that involved multi-sectoral departments such as key education providers, policy makers, and local Aboriginal community-controlled organisations. The education program, facilitated via a Tertiary and Further Education (TAFE) institution, offered experiential learning with participants (*n* = 21) working together as a team, facilitated by a mentor. Outcomes were assessed using a 25-item knowledge test and a confidence survey with three subscales (knowledge, skills, communication), with statements assessed using a Likert-type scale (number of items not reported). Four course participants did not participate in the evaluation (reasons not given), resulting in a small sample size (*n* = 17). The authors reported that participants increased their CVD knowledge (*p* <  0.001) and confidence in their CVD knowledge, skills, and communication (*p* <  0.001). However, statistical differences in the latter scores were assessed using *t*-tests, which should be reserved for use with normally distributed scale data. The involvement of various partners had a positive impact by exposing participants to different clinical situations and learning opportunities. This collaborative approach to education facilitated increased cultural competence and expertise in the delivery of cardiovascular services to Indigenous communities.

Three studies investigated rehabilitation programs [33, 35, 37]. One study was conducted over an eight-week period within a cardiopulmonary rehabilitation centre in Tasmania [33]. The program was managed by Aboriginal community controlled health services, and included peer support from an Indigenous health worker. Recommendations from family and friends enabled new participants to join. Indigenous participants were provided with easy access via provision of transport services. The program was comprised mainly of supervised exercise and education sessions, with the latter focused on cardiovascular and respiratory health and disease, self-management, benefits of exercise, nutrition, medication usage, stress management and psychological well-being, and smoking cessation. These aspects of intervention were directed towards both prevention and maintenance of health behaviours. The outcome measures included participation level, development of positive health behaviours, and improved general health with improved exercise tolerance. Although there was 22% loss to follow up, resulting in a relatively small sample size (*n* = 72), implementation of this program resulted in “clinically significant” improvements in several measures of cardiovascular health. Reductions in weight loss, waist circumference, and BMI were demonstrated, but were reported as statistically non-significant, although some cited effect sizes were medium to large. As well, Six Minute Walk Test distance, Incremental Shuttle Walk Test distance, and Timed Up and Go Test time were all increased, but were not statistically significant. Quality of life was also improved in most domains of the SF-36; although improvements were reported as “statistically significant” no values were cited. Success was attributed to a well-designed, one-stop-shop. The program had a holistic approach that addressed several health issues such as nutrition, exercise, smoking cessation and medication management. The program also addressed issues of cultural safety by conducting the study in an Aboriginal community controlled health service.

The study by Daws et al. [37] implemented a “working together” model of care in which an Aboriginal Hospital Liaison Officer and a specialist cardiac nurse teamed up to address the problem of referring patients to rehabilitation services post-acute care. The Aboriginal Hospital Liaison Officer made the initial contact with all patients and facilitated their meeting with the cardiac nurse. This introduction was vital for establishing relationships. Together, the partnership arranged referrals and provided education about the benefits of cardiac rehabilitation, and helped improve access to cardiac rehabilitation health services for the Indigenous participants. Retrospective medical chart audits revealed that the model improved referrals from 15 to 86% and rehabilitation attendance rates from zero to 62%. However, it is important to note that pre-implementation data were collected over a 3-year period (*n* = 68), whereas post-implementation data (*n* = 15) were collected over a 7-month period, and no comparisons were made between the two cohorts. Thus, it is difficult to draw conclusions about the effectiveness of this project. In a similar project, within an Aboriginal medical service, Dimer et al. [35] aimed to provide a secure environment for an eight-week exercise and education program for Aboriginal people with or at risk of CVD. Both visual and experiential learning opportunities were employed. Education and exercise through yarning helped to identify and address a range of issues such as medication compliance, risk factor reviews, and chest pain management. The cardiac rehabilitation program was well attended (*n* = 98), but only half of the participants (49%, *n* = 48) was surveyed (reasons not given). Of these, outcome measures were reported on 28 participants that completed the 8-week program only, thus it is difficult to evaluate overall effectiveness of the program as the outcome data reported is representative of only 29% of all program participants. In the program-completion subset, statistically significant reductions in body mass index (*p* <  0.05), waist girth (*p* <  0.01), systolic (*p* <  0.01) and diastolic (*p* <  0.05) blood pressure, and increased 6-Minute Walk Test distance (*p* <  0.01), were reported. Success of the program was attributed to the delivery of the program in a culturally safe environment. The researchers concluded that rehabilitation programs for Indigenous people were more effective when they were planned as part of an already established service. The program enabled local community members to be involved in the planning of activities.

## Discussion

The aim of this review was to systematically examine the published literature for evidence of the effectiveness of cardiovascular health interventions designed for Indigenous Australians. Eight studies were identified that met the inclusion criteria, demonstrating the dearth of formal research in this area.

There was considerable heterogeneity within the studies that were reviewed with interventions implemented in a wide variety of clinical settings across Australia, and measuring different outcomes. Thus, a meta-analysis was not possible. In order to examine the effectiveness of the interventions, there is a need for further experimental research to demonstrate objectively the effects of cardiovascular programs in terms of health outcomes as well as Indigenous engagement and satisfaction.

Although there was only a relatively small number of intervention studies available for review, the overall results indicate that targeted cardiovascular programs are effective in improving clinical outcomes, such as weight reduction, blood pressure control, and increased activity levels. As well, several programs demonstrated behavioural impacts regarding healthy lifestyle changes, and provided valuable insights into successful strategies for Indigenous people that may help to inform future programs. Indigenous people display cardiovascular symptoms at a younger age compared to non-Indigenous people [1]. However, poor screening of young Indigenous people has been reported [23], suggesting a need for earlier targeted assessment and advice about CVD risk factors, commencing during school years.

Although much higher rates of cigarette smoking have been reported for Indigenous Australians [1], intervention programs addressing this risk factor in association with CVD were not found in this review. Similarly, findings by Arjunan and colleagues [42] revealed the scarcity of local evidence crucial for promoting cessation among Aboriginal tobacco smokers. The risk to health from smoking and alcohol is widely acknowledged. In particular, smoking impacts cancer, respiratory, and cardiovascular diseases [1] whereas Indigenous alcohol use is primarily associated with impacts on mental health [1].

In the main, it was observed that the reviewed programs were designed within a mainstream health model with somewhat limited consideration given to the perspectives of Indigenous patients, their families or communities, suggesting that there is a cultural sensitivity gap in the design of cardiovascular interventions. Culturally sensitive health care has been described as care that effectively responds to the attitudes, feelings, and circumstances of individuals that belong to a population group with common identifying characteristics such as race, religion, language, and socioeconomic status, and that which patients perceive as being concordant with their cultural values and beliefs [43]. Cultural sensitivity is, therefore, the extent to which ethnic or cultural characteristics, experiences, norms, values, behavioural patterns and beliefs of the target population as well as relevant historical, environmental, and social forces are incorporated into the design, delivery, and evaluation of targeted health programs [44]. In 2016, the World Federation of Critical Care Nurses made a declaration about culturally sensitive practice [45]. Its recommendations, whilst directed at critical care nurses, are applicable to many settings. Included are aspects such as self-assessment by clinicians, establishing trust with patients and their families, identification of patients’ culture (language, food, gender considerations), and ensuring that dignity and privacy are protected.

Since the focus of the review was on the Indigenous population, each study was reviewed in this context by examining its aims and objectives, its focus on Indigenous participation, and particular Indigenous-sensitive aspects reflected in the design and implementation of its CVD program: access, empowerment, collaborative partnerships, and meaningful relationships. Following their 2012 study on effects of exercise and nutrition, Canuto et al. [46] went a step further by evaluating their program qualitatively. A main finding was that participants’ perception of health benefits of the program influenced their attendance. They concluded that programs should be designed to meet participants’ needs and expectations, and be conducted in a culturally safe environment. Healthcare providers should commit to the process of building and maintaining cultural relationships [47]. Neuwelt and colleagues, [48] in their study of the role of receptionists in general practice settings, revealed the importance of creating meaningful relationships with Indigenous patients. The receptionists helped patients to feel comfortable, demonstrating that trustworthiness and healthy relationships with patients are important factors that encourage sustained Indigenous engagement. Similarly, Hayman and colleagues [49] pointed to the fact that successful implementation of programs at a community controlled health centre was partly as a result of employment of Indigenous staff, good interpersonal relationships, and effective collaboration and consultation processes with the Indigenous community.

In several studies, Indigenous access was enhanced by use of local community based services [31, 35, 36, 38] providing a one-stop-shop type of service [38]. Further, transportation was offered for those that needed it, enabling access for those living in more remote areas [33, 34]. A study by Tuttle, et al. [50] made comparison between Indigenous and non-Indigenous participants in an outreach program to measure completion of scheduled outreach visits of the intervention group. An interesting finding was that distance alone did not influence completion of visits. Although Indigenous participants resided further away from the hospital in comparison to non-Indigenous participants, the study revealed that there was no difference in the number of attempted and missed visits at baseline. A combination of distance and timing of the visits had a significant impact in that at six months visits there were more missed appointments. A similar pattern was also reflected in the study by Artuso et al. [51] who investigated the factors that influenced utilisation of health care among Indigenous cardiac participants. They found that the perceived need for the service influenced long term utilisation. Patients that felt they were ‘fixed’ or cured after a procedure, or in this case a program, they did not see the need to continue with treatment. The authors concluded that it was not sufficient to only provide a program but to understand the needs of individuals; emphasising to participants that health maintenance is a lifelong commitment.

Indigenous empowerment was enhanced both at individual and community levels, through use of individualised care plans [32, 36, 38], training of Indigenous workers [34, 37], and involvement of community advisory committees [31, 35, 37], and ‘working together’ approaches between multidisciplinary teams developed meaningful collaborative partnerships [31, 34–36, 38]. Multidisciplinary teams allowed for focused use of approaches that addressed Indigenous needs and team learning allowed for a better work environment [33, 38]. Continuity of care was possible with teamwork approaches [31, 35, 37, 38] and relationships that were appropriate and culturally safe were achieved through involvement of participants and their community [34, 35, 37]. Providing time to understand participants through yarning and other communication styles that are sensitive to Indigenous people contributed to a culturally safe environment [33–35, 37, 38]. Common components associated with intervention effectiveness were: integration and coordination of programs within the existing services, such as metropolitan areas [31], primary health services [32, 36, 38], Aboriginal medical services [33, 35], active involvement of Indigenous health workers in the form of education and clinical partnerships with other health care providers [34, 37], and provision of support to facilitate individual participation through transport provision, peer/family support, and professional support [33, 38].

Our results indicate that an Indigenous perspective was crucial to not only deliver apposite strategies, but to ameliorate the subtle negative impact that colonisation exerts on Indigenous people, reminiscent of the initial forced changes in lifestyle, familial and cultural practices, and spiritual disconnectedness which affect Indigenous people in complex ways [14, 52]. Indigenous engagement was recommended in the studies, where findings illustrated that whilst the distance from health services influenced Indigenous access, there were other culturally rooted factors that contributed to success and required negotiation of meeting times and locations with the patients. Similar factors were reflected in a narrative report of an Indigenous cardiac outreach program in which success was attributed to integration of Indigenous values, which encouraged capacity building, and ownership of programs by either individuals or communities [21]. Our results suggest there is a clear need to employ healthcare strategies that incorporate traditional knowledge, and reflect Indigenous values. Without appropriate cultural and contextual knowledge, participants may hold mistaken beliefs about their health outcome, leading to a failure of some participants to engage.

The continued use of mainstream healthcare models displays subtle patronising implications, where traditional knowledge is not adequately acknowledged nor utilised when addressing health issues. Valuing and integrating Indigenous wisdom into the design of healthcare programs can enhance therapeutic relationships between Indigenous communities and health services by uniting and empowering both Indigenous and non-Indigenous peoples as contributors to health [53, 54]. The key to effectiveness of collaborative partnerships lies in genuine involvement of Indigenous people and validation of traditional practices and influences on policy [55]. For example, ‘working together’ programs in multidisciplinary teams have shown demonstrable benefits for participants [34, 37]. Such programs provide opportunities for learning and create a respectful and reciprocal relationship through genuine understanding by incorporating Indigenous worldviews and cultural preferences [56]. However, whilst cultural sensitivity is important in the prevention and treatments of behavioural ailments caution has been expressed [55, 57] that it should not be used as a buzz word or in a tokenistic way.

Although there was only a relatively small number of intervention studies available for review, the overall results indicate that targeted cardiovascular programs are effective in improving clinical outcomes, such as weight reduction, blood pressure control, and increased activity levels. As well, several programs demonstrated behavioural impacts regarding healthy lifestyle changes, and provided valuable insights into successful strategies for Indigenous people that may help to inform future programs.

### Implications for indigenous health care services

The results of this review provide a clear indication of the importance of Indigenous inclusivity and cultural sensitivity when implementing programs aimed at improving Indigenous CVD outcomes. To make a difference in improved health outcomes for this population group, sustainable interventions and continued development of new models of care that meet and manage Indigenous peoples’ health needs is critical. This has implications for healthcare professional training and education, which should serve to enhance understanding of differences in individuals that seek health care, especially Indigenous people, emphasising the importance of constructing meaningful relationships and ‘working together’ approaches. This responsibility transcends departmental boundaries to the general public. For effective programs to be implemented and sustained, rigorous research methods and appropriate programs that are responsive to Indigenous issues and needs will enhance change and impact Indigenous health outcomes positively.

### Limitations

There were two key limitations to this review. Of particular note was the lack of randomised trials, which would have provided stronger evidence of the effectiveness of interventions. As well, there was significant heterogeneity of settings, interventions and outcomes in the studies that were reviewed, making generalisation difficult. Despite these limitations, some clear themes were evident regarding Indigenous inclusivity and its association with successful outcomes, including both physical and behavioural components.

## Conclusions

There are very few studies that have investigated the effectiveness of cardiovascular health interventions designed to address Indigenous health outcomes. Further rigorous evaluation would enable a better understanding of effectiveness and sustainability of cardiovascular programs among Indigenous Australians. Nonetheless, the reviewed interventions have demonstrated a range of tangible benefits and provided insight into factors that contribute to the success of such programs. Our results suggest that healthcare professionals should actively incorporate the values of Indigenous people into the design of cardiovascular healthcare programs, demonstrating respect and reciprocity through meaningful collaboration with Indigenous people. Clearly the synergy of multidisciplinary teams and collaborative partnerships benefits both patients and health staff alike in a way that can only advance cardiovascular health for Indigenous Australians.

## Abbreviations

BMI: Body mass index
COAG: Council of Australian Governments
CVD: Cardiovascular disease
JBI: Joanna Briggs Institute
PEPI: Program for epidemiologists
TAFE: Technical and Further Education

## References

[1] Australian Institute of Health and Welfare. Australian Burden of Disease Study: Impact and Causes of Illness and Death in Aboriginal and Torres Strait Islander People 2011. Australian Burden of Disease Study series no. 6. Cat. no. BOD 7. Canberra, ACT: Australian Institute of Health and Welfare, 2016. Available from: https://www.aihw.gov.au/getmedia/e31976fc-adcc-4612-bd08-e54fd2f3303c/19667-bod7-atsi-2011.pdf.aspx?inline=true. Accessed 19 Sept 2018.

[2] Australian Institute of Health and Welfare. Cardiovascular disease. Australian facts 2011. Cardiovascular disease series. Cat. no. CVD 53. Canberra, ACT: Australian Institute of Health and Welfare, 2011. Available from: https://www.aihw.gov.au/getmedia/13cd081b-8123-4660-ad68-3d780c12ffeb/12116-20111005.pdf.aspx. Accessed 19 Sept 2018.

[3] Council of Australian Governments (2008). Closing the gap in indigenous disadvantage. National Integrated Strategy for closing the gap in indigenous disadvantage. Canberra. ACT: Council of Australian Governments.

[4] World Health Organisation. Cardiovascular diseases. Fact sheet no. 317. Geneva: World Health Organisation, 2013. Available from: http://www.who.int/mediacentre/factsheets/fs317/en/. Accessed 19 Sept 2018.

[5] Council of Australian Governments Reform Council (2014). Healthcare in Australia 2012-13: comparing outcomes by indigenous status. Supplement to the reports to the Council of Australian Governments. Sydney. NSW: Council of Australian Governments Reform Council.

[6] Australian Institute of Health and Welfare. Australia’s Health 2014. Australia’s health series no. Cat. No. AUS 178. Canberra, ACT: Australian Institute of Health and Welfare, 2014. Online. Available from: http://www.aihw.gov.au/publication-detail/?id=60129547205. Accessed 19 Sept 2018.

[7] Manuel DG, Tuna M, Hennessy D, Bennett C, Okhmatovskaia A, Fines P (2014). Projections of preventable risks for cardiovascular disease in Canada to 2021: a microsimulation modelling approach. CMAJ Open.

[8] World Health Organisation. Cardiovascular Diseases (CVDs). Fact sheet. Geneva, Switzerland: World Health Organisation, 2017. Available from: http://www.who.int/mediacentre/factsheets/fs317/en. Accessed 19 Sept 2018.

[9] United Nations. State of the World’s indigenous peoples. New York, NY: Department of Economic and Social Affairs, 2009. Available from: https://www.un.org/esa/socdev/unpfii/documents/SOWIP/en/SOWIP_web.pdf. Accessed 19 Sept 2018.

[10] Vos T, Barker B, Begg S, Stanley L, Lopez AD (2009). Burden of disease and injury in Aboriginal and Torres Strait Islander peoples: the Indigenous health gap. Int. J. Epidemiol.

[11] Australian Health Minister’s Advisory Council (2012). Aboriginal and Torres Strait islander health performance framework 2012 report.

[12] Australian Bureau of Statistics. Causes of death 2016 (3303.0). September 2017.

[13] Australian Institute of Health and Welfare (2017). National Key Performance Indicators for Aboriginal and Torres Strait Islander Primary Health Care: Results from June 2016. National key performance indicators for aboriginal and Torres Strait islander primary health care series no. 4. Cat. No. IHW 177.

[14] King M, Smith A, Gracey M (2009). Indigenous health part 2: the underlying causes of the health gap. Lancet.

[15] Eckermann A, Dowd T, Chon E, Nixon L, Gray R. Binan Goonj: Bridging Cultures in Aboriginal Health. 3rd ed. Sydney, NSW: Elsevier; 2010. Available from: http://www.healthinfonet.ecu.edu.au/key-resources/bibliography/?lid=19201. Accessed 19 Sept 2018.

[16] Ride K, Thomson N (2010). Summary of indigenous health: cardiovascular health status. Aboriginal Islander Health Worker J.

[17] Fuster V, Kelly BB (2010). Institute of Medicine. Promoting cardiovascular health in the developing world: a critical challenge to achieve Global Health.

[18] Lloyd-Jones DM, Hong Y, Labarthe D, Mozaffarian D, Appel LJ, Van Horn L (2010). Defining and setting national goals for cardiovascular health promotion and disease reduction: the American Heart Association's strategic Impact Goal through 2020 and beyond. Circulation.

[19] Jackson SF, Perkins F, Khandor E, Cordwell L, Hamann S, Buasai S (2006). Integrated health promotion strategies: a contribution to tackling current and future health challenges. Health Promot. Int.

[20] Grace SL, Angevaare KL, Reid RD, Oh P, Anand S, Gupta M (2012). Effectiveness of inpatient and outpatient strategies in increasing referral and utilization of cardiac rehabilitation: a prospective, multi-site study. Implement Sci.

[21] Tibby D, Corpus R, Walters DL (2010). Establishment of an innovative specialist cardiac Indigenous outreach service in rural and remote Queensland. Heart Lung Circ.

[22] Ski Chantal F, Vale Margarite J, Bennett Gary R, Chalmers Victoria L, McFarlane Kim, Jelinek V Michael, Scott Ian A, Thompson David R (2015). Improving access and equity in reducing cardiovascular risk: the Queensland Health model. The Medical Journal of Australia.

[23] Thompson SC, Haynes E, Woods JA, Bessarab DC, Dimer LA, Wood MM (2016). Improving cardiovascular outcomes among Aboriginal Australians: Lessons from research for primary care. SAGE Open Med.

[24] Joanna Briggs Institute. Reviewers’ Manual 2014 Edition. The University of Adelaide, South Australia 2014. Available from: http://joannabriggs.org/assets/docs/sumari/ReviewersManual-2014.pdf. Accessed 19 Sept 2018.

[25] The Cochrane Collaboration. Glossary of Terms in the Cochrane Collaboration, Version 4.2.5 [updated May 2005]. Chichester, UK: The Cochrane Collaboration; 2005. Available from: aaz.hr/resources/pages/57/7.%20Cochrane%20glossary.pdf (accessed 22 March 2018).

[26] Gough D, Oliver S, Thomas J (2012). An introduction to systematic reviews.

[27] Mbuzi V, Fulbrook P, Jessup M. Effectiveness of strategies used in the management of cardiac conditions among Indigenous Australians. Study protocol. PROSPERO; 2016: CRD42016046688 Available from: http://www.crd.york.ac.uk/PROSPERO/display_record.php?ID=CRD42016046688. Accessed 19 Sept 2018.

[28] The Joanna Briggs Institute. JBI data extraction form for experimental/observational studies. The University of Adelaide, South Australia. 2014. Available from: https://joannabriggs.org/assets/docs/jbc/operations/dataExtractionForms/JBC_Form_DataE_ExpObs.pdf. Accessed 19 Sept 2018.

[29] The Joanna Briggs Institute. Critical appraisal tools. The University of Adelaide, SA: The Joanna Briggs Institute; 2016. Available from: https://joannabriggs.org/research/critical-appraisal-tools.html. Accessed 19 Sept 2018.

[30] Reilly R, Evans K, Gomersall J, Gorham G, Peters MD, Warren S (2016). Effectiveness, cost effectiveness, acceptability and implementation barriers/enablers of chronic kidney disease management programs for Indigenous people in Australia, New Zealand and Canada: a systematic review of mixed evidence. BMC Health Serv. Res.

[31] Canuto KJ, Cargo M, Li M, D’Onise K, Esterman A, McDermott RA. Pragmatic randomised trial of a 12 week exercise and nutrition program for aboriginal and Torres Strait women: clinical results immediate post and 3 months follow-up. BMC Public Health. 2012;12(933) http://www.biomedcentral.com/1471-2458/12/933. Accessed 19 Sept 2018. 10.1186/1471-2458-12-933 PMC3608991 23114379

[32] Peiris D, Usherwood T, Panaretto K, Harris M, Hunt J, Redfern J (2015). Effect of a computer-guided, quality improvement program for cardiovascular disease risk management in primary health care the treatment of cardiovascular risk using electronic decision support cluster randomized trial. Circ. Cardiovasc. Qual. Outcomes.

[33] Davey M, Moore W, Walters J. Tasmanian aborigines step up to health: evaluation of a cardiopulmonary rehabilitation and secondary prevention program. BMC Health Serv Res. 2014;14(349) http://www.biomedcentral.com/1472-6963/14/349. Accessed 19 Sept 2018. 10.1186/1472-6963-14-349 PMC4141095 25134693

[34] Davidson PM, DiGiacomo M, Abbott P, Zecchin R, Heal PE, Mieni L (2008). A partnership model in the development and implementation of a collaborative, cardiovascular education program for Aboriginal health workers. Aust. Health Rev.

[35] Dimer L, Dowling T, Jones J, Cheetham C, Thomas T, Smith J (2013). Build it and they will come: outcomes from a successful cardiac rehabilitation program at an Aboriginal Medical Service. Aust. Health Rev.

[36] Burgess CP, Sinclair G, Ramjan M, Coffey PJ, Connors CM, Katekar LV (2015). Strengthening cardiovascular disease prevention in remote Indigenous communities in Australia’s Northern Territory. Heart Lung Circ.

[37] Daws K, Punch A, Winters M, Posenelli S, Willis J, MacIsaac A (2014). Implementing a working together model for Aboriginal patients with acute coronary syndrome: An Aboriginal Hospital Liaison Officer and a specialist cardiac nurse working together to improve hospital care. Aust. Health Rev.

[38] Burgess CP, Bailie RS, Connors CM, Chenhall RD, McDermott RA, O’Dea K (2011). Early identification and preventive care for elevated cardiovascular disease risk within a remote Australian Aboriginal primary health care service. BMC Health Serv. Res.

[39] Higgins JPT, Altman DG, Gøtzsche PC, Jüni P, Moher D, Oxman AD (2011). Cochrane Bias Methods Group, Cochrane Statistical Methods Group. The Cochrane Collaboration’s tool for assessing risk of bias in randomised trials. BMJ.

[40] Omura M, Maguire J, Levett-Jones T, Stone TE (2017). The effectiveness of assertiveness communication training programs for healthcare professionals and students: A systematic review. Int. J. Nurs. Stud.

[41] The Joanna Briggs Institute Levels of Evidence and Grades of Recommendation Working Party. New JBI Levels of Evidence. The Joanna Briggs Institute. The University of Adelaide, South Australia. 2013. Available from: https://joannabriggs.org/assets/docs/approach/JBI-Levels-of-evidence_2014.pdf. Accessed 19 Sept 2018.

[42] Arjunan P, Poder N, Welsh K, Bellear L, Heathcote J, Wright D (2016). Smoking among Aboriginal adults in Sydney, Australia. Health Promotion Journal of Australia.

[43] Tucker Carolyn M., Arthur Tya M., Roncoroni Julia, Wall Whitney, Sanchez Jackeline (2013). Patient-Centered, Culturally Sensitive Health Care. American Journal of Lifestyle Medicine.

[44] Singelis TM, Garcia RI, Barker JC, Davis RE (2018). An experimental test of the two-dimensional theory of cultural sensitivity in health communication. J Health Commun.

[45] Friganovic A, Bloomer M, Northam H, Kalauz S, Zellinger M, Lopez V, Fulbrook P (2016). World Federation of Critical Care Nurses: Brisbane declaration on culturally sensitive critical care nursing. Connect: The World of Critical Care Nursing.

[46] Canuto Karla J, Spagnoletti Belinda, McDermott Robyn A, Cargo Margaret (2013). Factors influencing attendance in a structured physical activity program for Aboriginal and Torres Strait Islander women in an urban setting: a mixed methods process evaluation. International Journal for Equity in Health.

[47] Davy C, Cass A, Brady J, DeVries J, Fewquandie B, Ingram S (2016). Facilitating engagement through strong relationships between primary healthcare and Aboriginal and Torres Strait Islander peoples. Aust NZ J Public Health.

[48] Neuwelt PN, Kearns RA, Cairns IR (2016). The care work of general practice receptionists. J. Prim. Health Care.

[49] Hayman N (2010). Strategies to improve Indigenous access for urban and regional populations to health services. Heart Lung Circ.

[50] Tuttle CS, Carrington MJ, Stewart S, Brown A (2016). Overcoming the tyranny of distance: An analysis of outreach visits to optimise secondary prevention of cardiovascular disease in high-risk individuals living in Central Australia. Aust. J. Rural Health.

[51] Artuso S, Cargo M, Brown A, Daniel M (2013). Factors influencing health care utilisation among aboriginal cardiac patients in Central Australia: a qualitative study. BMC Health Serv Res.

[52] Gray C, Brown A. Thomson N. Australian Indigenous HealthInfoNet: Review of cardiovascular health among Indigenous Australians; 2012. Available from: http://www.healthinfonet.ecu.edu.au/chronic-conditions/cvd/reviews/heart_review. Accessed 19 Sept 2018.

[53] Osborne K, Baum F, Brown L. What works? A review of actions addressing the social and economic determinants of indigenous health. Issues paper no. 7. Australian Institute of Health and Welfare, Australian Institute of Family Studies: Canberra, ACT; 2013. Available from: www.healthinfonet.ecu.edu.au/uploads/resources/26675_26675.pdf. Accessed 19 Sept 2018.

[54] Stephens C, Porter J, Nettleton C, Willis R (2006). Disappearing, displaced, and undervalued: a call to action for Indigenous health worldwide. Lancet.

[55] Lucero E (2011). From tradition to evidence: decolonization of the evidence-based practice system. J. Psychoactive Drugs.

[56] Wilson D (2008). The significance of a culturally appropriate health service for Indigenous Maori women. Contemp. Nurse.

[57] Williams E, Guenther J, Arnott A. Traditional healing: a literature review. Working paper series 2: evaluation and policy. No. 2. Co-valuator Network: Alice Springs, NT; 2011. Available from: www.covaluator.net/docs/S2.2_traditional_healing_lit_review.pdf. Accessed 19 Sept 2018.

